# A telomere-to-telomere genome assembly of the protandrous hermaphrodite blackhead seabream, *Acanthopagrus schlegelii*

**DOI:** 10.1038/s41597-025-04602-y

**Published:** 2025-02-27

**Authors:** Kai Zhang, Sixin Guo, Shaosen Yang, Wenchuan Zhou, Jinhui Wu, Xinhui Zhang, Qiong Shi, Li Deng

**Affiliations:** 1 Laboratory of Aquatic Genomics, College of Life Sciences and Oceanography, Shenzhen University, Shenzhen, 518057 China; 2 Shenzhen Key Lab of Marine Genomics, Guangdong Provincial Key Lab of Molecular Breeding in Marine Economic Animals, BGI Academy of Marine Sciences, Shenzhen, 518081 China; 3 Agro-Tech Extension Center of Guangdong Province, Guangzhou, 510225 China; 4 Shenzhen Fishery Development Research Center, Shenzhen, 518067 China

**Keywords:** Comparative genomics, Comparative genomics

## Abstract

A remarkable life cycle of the protandrous blackhead seabream (*Acanthopagrus schlegelii*), initiating as a male during the first two years and then naturally transforming to a female since the third year, makes this fish a valuable model for studying molecular mechanisms of sex change. Here, we constructed a gap-free telomere-to-telomere (T2T) genome assembly for a male blackhead seabream, by integration of PacBio HiFi, Ultra-long ONT and Hi-C sequencing techniques. With 97.87% of the entire sequences anchored into 24 chromosomes, this haplotypic genome assembly spans 714.98 Mb. In terms of correctness (quality value QV: 52.95) and completeness (BUSCO score: 99.9%), this chromosome-scale assembly is indeed of high quality. It has been annotated with 24,581 protein-coding genes, and predicted with low percentage (30.95%) of repetitive sequences. As the first reference T2T-level genome assembly of various protandrous fishes, it provides a valuable genetic resource for expansion of fish genomics database. It will also allow for in-depth genomic comparisons among diverse hermaphrodite vertebrates, as well as offer fundamental genome data to support extensive research on blackhead seabream.

## Background & Summary

Belonging to the order Perciformes and the family Sparidae, blackhead seabream (*Acanthopagrus schlegelii*) has been among the most economically important and well-liked marine fish species. It has quick development, outstanding flesh quality, and good environmental adaptability^1,2^. Its artificial breeding and aquaculture is expanding quickly as a result of the growing demand in the market. Implementing artificial breeding or selection has been employed to facilitate the increased production of blackhead seabream. Sex control is therefore applied as one of the most crucial strategies.

Interestingly, blackhead seabream is a protodromous hermaphrodite with an impressive life cycle of natural sex change from male to female, making it a valuable model for studying molecular mechanisms of fish sex development^3^.The fish exhibit sexual differentiation during its juvenile stage, possessing a bisexual gonad. This can lead to a fact that male and female reproductions are not synchronized and the ratio of female to male is severely disproportional. Without the ability to regulate sexual differentiation, maturation, and reproduction, farmers indeed have little control over practical breeding processes. Interestingly, previous studies showed that age and season play an combined impact on the dynamic process of bisexual gonad development^3^. Meanwhile, development of vitellogenic oocytes in the ovary serves as one of the indicators for natural sex change in blackhead seabream^4,5^. Although some genes such as *wnt4* (Wnt family member 4), *foxl2* (Forkhead Box L2)*, cyp19a1a* (cytochrome P450, family 19, subfamily A, polypeptide 1a)*, dmrt1* (doublesex and mab-3 related transcription factor 1)*, amh* (anti-Mullerian hormone), and *amhr2* (anti-Mullerian hormone receptor type 2), potentially related to sexual differentiation and sex controlling, have been reported^1,4,5^; however, the detailed molecular mechanisms of natural sex change is still unknown in blackhead seabream.

For investigations of functional, ecological, and evolutionary genomics in this species as well as other hermaphrodite fishes, it is necessary for researchers to have a well-assembled genome in hand. A draft genome assembly of a female blackhead seabream was released by us in 2018, containing 89% of the full BUSCOs (actinopterygii_odb9 database) and numerous contigs (115, 091) with a low N50 value of 17.2 kb^6^. Although this assembly provided an important genetic resource for comparative genomics studies on various Perciformes, its fragmentation and incompleteness has restricted its comprehensive applications in fish research. In our present work, we assembled a gap-free telomere-to-telomere (T2T) genome of a male blackhead seabream by integration of PacBio (Pacific Biosciences) HiFi long reads, ONT (Oxford Nanopore Technologies) ultra-long reads, MGI short reads, and Hi-C (High-through chromosome conformation capture) sequencing reads. This high-quality genome dataset will greatly enable further works on understanding of biological characteristics (especially sex change) of blackhead seabream.

## Methods

### Sample collection

**Fig. 1:**
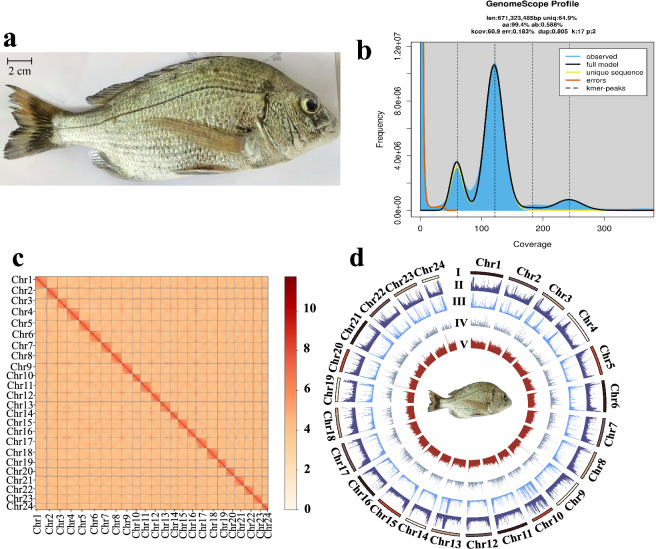
A T2T genome assembly of the protandrous hermaphrodite blackhead seabream. **(a)** A photo of the sequenced fish. **(b)** A GenomeScope k-mer plot. **(c)** A total of 24 distinct blocks in the Hi-C contact matrixes. (**d**) A Circos plot of the main genome features. From outside to inside: (I) the 24 chromosomes, (II) repeats, (III) Long terminal repeat (ltr), (IV) gene density, and (V) GC content. Links inside the Circos refer to internal syntenic blocks among different chromosomes within the assembled genome.

A two-year-old male adult blackhead seabream (Fig. 1a) was collected from Guangdong Marine Fisheries Experimental Centre, which belongs to the Agro-Tech Extension Center of Guangdong Province with an offsite location in Huizhou city, Guangdong province, China. Muscle tissue was pooled for whole genome sequencing, and muscle, liver, brain, gill, and gonad were collected for additional transcriptome sequencing. The sampling procedure and practical pipeline were conducted according to the recommendations and approval of the Animal Ethics Committee of Shenzhen University (Shenzhen, China; license number: A202301455).

### DNA extraction and genome sequencing

A blood & cell culture DNA kit (Qiagen, USA) was used for extraction and purification of genomic DNA (gDNA) from the muscle in accordance with the manufacturer’s instructions. The extracted gDNA was used for construction of a library (insert size of 350 bp) with the MGIEasy Universal DNA Library Prep Kit (MGI, China), which was then sequenced on an MGISEQ. 2000 platform (MGI). A total of 100.47 Gb of raw reads (150 bp in length) were generated, among them low-quality reads and adaptor sequences were filtered using SOAPfilter (v2.2)^7^ with default settings. Finally, we obtained 95.98 Gb of clean reads for estimating genome size and assembling sequences.

Additionally, long-read libraries were created utilizing a PacBio Sequel II System and a SMRTbell Express Template Prep Kit 2.0 for HiFi sequencing, following PacBio’s standard technique (Pacific Biosciences, USA). The consensus sequences were then produced using the CCS software (SMRT Link v9.0)^8^. Approximately 62.60 Gb of consensus reads with a mean length of 15.25 kb were obtained.

Oxford Nanopore Technologies (ONT) was applied for construction of an ultra-long library and then one flow cell was sequenced on a PromethION platform (Oxford Nanopore Technologies Co., UK). The raw reads were first refined to remove those with quality value (QV) below 7. Subsequently, Porechop (https://github.com/rrwick/Porechop) was applied to eliminate adaptors, and Filtlong (https://github.com/rrwick/Filtlong) was employed to filter out those reads shorter than 30 kb and mean read quality scores less than 90%. Finally, a total of 1.171 million clean reads were retained, accumulating a substantial count of 22.44 Gb. The average read length was 79.63 kb, with an N50 length of 100 kb.

For the Hi-C sequencing, DNA libraries were prepared with a GrandOmics Hi-C kit (GrandOmics, China; using DpnII as the restriction enzyme) as per the manufacturer’s instructions. The Hi-C libraries were then sequenced on an Illumina Novaseq system (Illumina, USA), generating 76.46 Gb of 150-bp paired-end raw reads. Trimmomatic (V0.39)^9^ with optimized parameters “SLIDINGWINDOW: 4:20, LEADING: 3, TRAILING:3, ILLUMINACLIP: adapter.fa:2:30:10:8:true” was used to filter out adaptor sequences, low-quality reads (quality scores < 20), and those reads shorter than 36 bp. This filtering process retained 63.53 Gb of clean data for subsequent construction of chromosomes.

### RNA extraction and transcriptome sequencing

Using a normal Trizol methodology (Invitrogen, USA), RNA samples were isolated from muscle, liver, brain, gill, and gonad tissues, and then purified using a Qiagen RNeasy Mini Kit (Qiagen). Following the manufacturer’s instructions, equivalent amount of RNA from each tissue were combined to create an Illumina cDNA library, which was subsequently sequenced on a HiSeq X Ten platform (Illumina). After generating 31.12 Gb of paired-end raw reads, adaptor sequences and low-quality reads were removed using SOAPfilter (v2.2)^7^ with default settings. In the end, 27.1 Gb of clean reads were retained for annotation of gene structures.

### Genome assembly

#### Genome-size estimation

Using Jellyfish (v2.2.6)^10^ and GenomeScope (v2.0)^11^, a K-mer frequency distribution of the MGI clean reads was determined. Accordingly, the genome size of blackhead seabream is calculated to be 671.32 Mb, and the rate of genomic heterozygosity was predicted to be 0.59% (Fig. 1b).

#### De novo genome assembly

In this study, HiFiasm (v0.19.5)^12^ was employed for assembling into contigs using HiFi + ONT long reads. Subsequently, these contigs were refined by T2T-polish^13^ with the optimized parameter set to task = best using the MGI short reads.This primary genome assembly was 731.09 Mb in length, and it is anchored into 41 contigs (with a contig N50 of 31.8 Mb).

#### Construction of chromosomes and gap filling

This high-quality genome assembly served as the foundation for subsequent construction of chromosomes using the Hi-C reads. Initially, Bowtie2 (v2.3.2)^14^ was employed to map clean reads to the primary genome assembly. Subsequently, the HiC-Pro (v2.8.1)^15^ pipeline was employed to detect valid contact paired reads. Using these Hi-C valid reads, the assembled contigs were anchored onto chromosomes via the 3D-DNA pipeline^16^ with the parameter set to -r 0. Manual correction of the chromosome-level scaffolds was then performed using JuiceBox (v1.11.08)^17^. Based on the HiFi and ONT long reads, we applied TGS-GapCloser (v1.1.1)^18^ with default parameters to resolve any remaining gaps in the chromosome-level genome assembly. As a result, we obtained the final genome assembly, which has a total size of 714.71 Mb and is anchored into 24 chromosomes with 97.78% of the primary assembly sequences (Fig. 1c, d and Table 1). Its contig N50 reaches 31.8 Mb.

**Table 1:** Telomere and centromere positions in the assembled blackhead seabream genome.

ID	Contigs	Length (bp)	Gaps	Centromere		Telomere			
				Start_pos	End_pos	Upstream start_pos	Upstream end_pos	Downstream start_pos	Downstream end_pos
Chr1	1	35,126,158	0	34,082,948	35,044,291	1	4,482	35,120,420	35,126,158
Chr2	1	33,180,304	0	32,746,417	33,114,509	1	6,233	33,176,476	33,180,304
Chr3	1	28,476,592	0	18,459,506	18,636,685	1	4,580	28,471,869	28,476,592
Chr4	1	37,352,144	0	7,985,027	8,469,646	1	3,553	37,347,922	37,352,144
Chr5	1	32,627,621	0	77,247	989,077	1	4,526	32,623,362	32,627,621
Chr6	1	34,863,683	0	34,201,854	34,765,318	1	4,570	34,857,447	34,863,683
Chr7	1	31,795,397	0	31,240,613	31,706,044	1	4,615	31,790,080	31,795,397
Chr8	1	32,478,475	0	32,070,863	32,435,351	1	3,716	32,473,311	32,478,475
Chr9	1	32,371,919	0	135,205	980,958	1	5,516	32,370,687	32,371,919
Chr10	1	24,772,937	0	15,209,740	15,424,643	1	3,576	24,771,480	24,772,937
Chr11	1	33,031,940	0	32,355,953	32,961,304	1	6,814	33,027,384	33,031,940
Chr12	1	27,619,938	0	22,486	414,489	1	2,639	27,615,689	27,619,938
Chr13	1	30,453,824	0	18,654,336	18,718,919	1	5,904	30,448,716	30,453,824
Chr14	1	23,346,931	0	97,742	578,166	1	5,627	23,340,274	23,346,931
Chr15	1	29,500,238	0	32,240	616,474	1	3,679	29,495,395	29,500,238
Chr16	1	26,722,964	0	26,224,990	26,645,641	1	4,647	26,717,638	26,722,964
Chr17	1	33,290,523	0	72,523	728,691	1	5,003	33,286,893	33,290,523
Chr18	1	32,865,489	0	329,793	636,170	1	5,694	32,862,016	32,865,489
Chr19	1	26,399,058	0	97,510	759,765	1	6,399	26,393,647	26,399,058
Chr20	1	25,860,688	0	356,948	917,343	NA	NA	25,856,389	25,860,688
Chr21	1	29,917,757	0	122,853	558,122	1	4,636	29,913,555	29,917,757
Chr22	1	27,516,636	0	69,126	477,565	1	4,996	27,516,078	27,516,636
Chr23	1	26,867,679	0	62,065	1,103,817	1	5,070	26,867,100	26,867,679
Chr24	1	18,536,763	0	74,986	1,196,278	1	3,912	18,532,396	18,536,763

#### Identification of centromere and telomere sequences

**Fig. 2:**
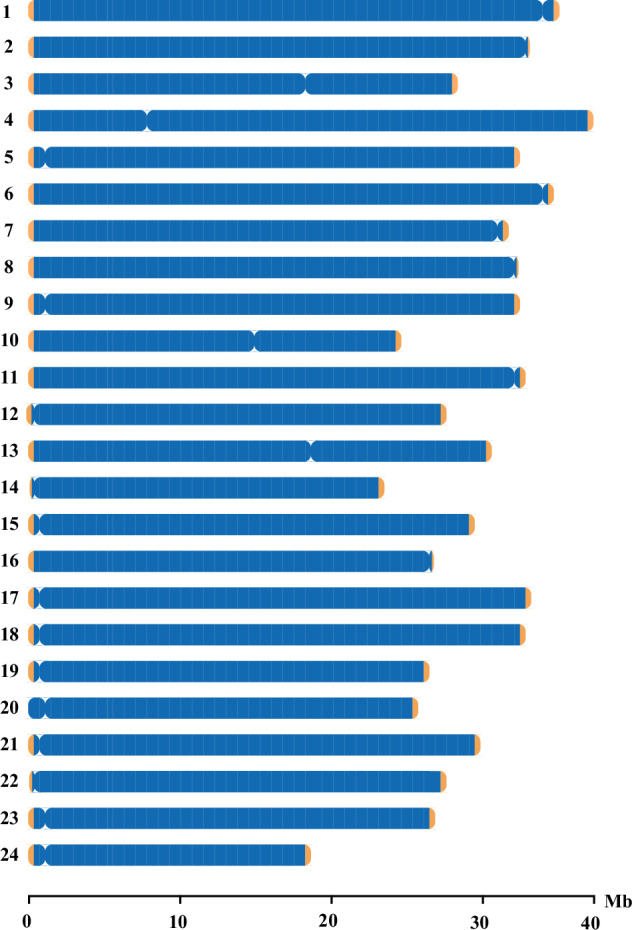
An overview of the T2T gap-free reference genome of blackhead seabream. The yellow areas at both ends of each chromosome represent the telomere regions, and the gully area within each chromosome represents the centromere region.

We identified telomere sequences through searching for repeating sequences (TTAGGG/CCCTAA) in telomeric regions. Centromere identification was performed by using the Centromics program (https://github.com/ShuaiNIEgithub/Centromics), which dealt with raw HiFi sequencing data, Hi-C sequencing data, and genome assembly data. Ultimately, we discovered that blackhead seabream chromosomes owned 24 centromeres and 47 telomeres (see more details in Fig. 2 and Table 1).

### Genome annotation

#### Repetitive sequence annotation

Both *ab initio* and homology-based strategies were employed to detect repetitive sequences in the blackhead seabream genome. In detail, using RepeatModeler (v2.0.1)^19^ and MITE-Hunter^20^ program with default settings, we constucted an *ab initio* repeat sequence library. This library was then aligned to the Repbase 24.0^21^ for classification of different repetitive elements via the TEclass tool^22^. For the homolog-based prediction based on the Repbase database^21^, DNA and protein transposable elements (TEs) were detected by RepeatMasker (v4.1.2) and RepeatProteinMasker (v4.0.7)^23^, respectively. Tandem repeats were identified with Tandem Repeat Finder (v4.10.0)^24^. After removal of redundant data from both methods, our results showed that a total of 221.23 Mb of repetitive sequences were identified, accounting for 30.95% of the assembled blackhead seabream genome (Table 2).

**Table 2:** Repetitive elements and their proportions in the assembled blackhead seabream genome.

Type	Repbase TEs		Protein TEs		Denovo TEs		Combined TEs	
	Length (bp)	Percentage (%)	Length (bp)	Percentage (%)	Length (bp)	Percentage (%)	Length (bp)	Percentage (%)
**DNA**	39,777,880	5.56	2,419,029	0.34	85,135,007	11.91	109,440,044	15.31
**LINE**	17,489,413	2.45	8,157,163	1.14	39,558,492	5.53	48,366,550	6.76
**SINE**	1,878,742	0.26	0	0	3,675,949	0.51	5,193,437	0.73
**LTR**	11,866,888	1.66	2,993,274	0.42	30,978,602	4.33	39,723,908	5.56
**Other**	10,810	0	0	0	0	0	10,810	0
**Unknown**	531,462	0.07	0	0	48,608,464	6.8	49,128,405	6.87
**Total**	64,716,941	9.05	13,567,398	1.9	187,505,428	26.23	221,275,390	30.95

#### Protein-coding genes and functional annotations

Three methods were combined to annotate protein-coding genes, including *ab initio* gene prediction, homology-based annotation, and transcriptome-based annotation. Using the HISAT2 (v2.2.1)^25^, refined transcriptome (RNA-seq) reads were aligned to the assembled genome for the transcriptome-based annotation. PASA^26^ was then applied to detect open reading frames (ORFs), and Stringtie^27^ was used for gene structure annotation by assembling corresponding transcripts. For the homology prediction, GeMoMa (v2.3)^28^ was employed to map protein sequences from four representative species (including yellowfin seabream *Acanthopagrus latus*, sharksucker *Echeneis naucrates*, zebrafish *Danio rerio*, and large yellow croaker *Larimichthys crocea*) to our assembly for prediction of gene structures. For the *ab initio* prediction, a training model library was constructed from a protein set generated from the RNA-seq reads by Trinity (v2.8.5)^29^. Augustus (v3.4.0)^30^ was then employed to annotate genes with the–noInFrameStop = true–strand = both setting based on the training data.

Integration of protein-coding genes annotated by these three approaches was carried out using the EVidenceModeler (EVM) pipeline (v1.1.1)^26^. Consequently, 24,081 protein-coding genes were identified, with an average gene length of 16.34 kb and an average coding sequence (CDS) length of 1,721.43 bp. The average number of exons per gene was 10.28, while exons averaged 271.91 bp in length and introns averaged 1,437.93 bp (see Table 3).

**Table 3:** Gene structures and the functional annotation.

Item	Number	Average length (bp)
Gene	24,581	16,343.87
Exon	10.28 (per gene)	271.91
Intro	**—**	1,437.93
**Database**	**Number**	**Percentage (%)**
InterPro	20,947	85.22
GO	14,501	58.99
KEGG_ALL	23,749	96.62
KEGG_KO	16,512	67.17
Swissprot	21,583	87.8
TrEMBL	23,684	96.35
NR	24,175	98.35

The protein-coding genes were functionally annotated by aligning them with several routine protein databases using Blastp (v2.2.26)^31^ and Diamond (v2.0.7)^32^. This alignment included comparisons with the NCBI nonredundant (NR) protein database (v5; released on September 29, 2020), as well as Swissprot (released on October 07, 2020)^33^, KEGG (released on October 1, 2019)^34^, TrEMBL (released on October 07, 2020)^34^, InterPro (v5.50–84.0)^35^ and Gene Ontology (GO; v1.2, released on July 27, 2023)^36^ databases. Of the total gene models, 24,179 (98.36%) were annotated with at least one homologous hit from these public databases (refer to Table 3).

#### Annotation of non-coding RNA genes

For annotation of non-coding RNA genes, tRNAscan-SE (v2.0.9)^37^ with default settings was employed to annotate tRNA-associated genes; rRNAs annotation was carried out using RNAmmer (v1.2)^38^; miRNAs and snRNAs were detected using Infernal (v1.1.2)^39^ against the Rfam (v1.4.1) database^40^ with default parameters. As a result, annotations predicted 956 rRNAs, 2,175 tRNAs, 874 miRNAs, and 1,212 snRNAs (see more details in Table 4).

**Table 4:** Statistics of the non-coding RNA annotations.

Type		Copy	Average length (bp)	Total length (bp)	% of genome
**miRNA**		874	87.58	7,6542	0.0107
**tRNA**		2,175	77.443	168,422	0.0236
**rRNA**	rRNA	956	196.55	187,901	0.0263
	18S	12	1,836	22,032	0.0031
	28S	13	4,582.46	59,572	0.0083
	5S	931	114.18	106,297	0.0149
**snRNA**	snRNA	1,212	151.31	183,383	0.0256
	CD-box	134	122.76	16,450	0.0023
	HACA-box	60	153.13	9,188	0.0013
	splicing	1,012	154.50	156,356	0.0219
	scaRNA	6	231.50	1,389	0.0002

## Data Records

All genome data have been uploaded to the NCBI SRA database with the BioProject accession PRJNA1134337, including the specific accessions SRR29908548 to SRR29908551^41–44^. The genome assembly have been deposited in the GenBank database under the accession number JBGQWW000000000^45^. Furthermore, detailed documents on genome assembly, gene structures, gene functions, and repeat annotations for blackhead seabream have been shared on the Figshare^46^.

## Technical Validation

### Evaluation of the genome assembly and annotation

To evaluate the quality of our genome assembly, we employed several approaches. First, we employed BUSCO (v5.2.2)^47^ to examine completeness. The BUSCO analysis revealed that, based on the 3640 single-copy orthologs in the actinopterygii_odb10 database, 99.3% of our annotated genes were correctly classified as complete (99.1% single-copy genes and 0.2% duplicated genes), with 0.6% being fragmented (see Table 5). Second, Merqury (v1.328)^48^ estimated the assembly quality value (QV) to be 52.95. Third, by aligning the sequencing data to the assembled genome, we evaluated the accuracy rate, which showed mapping rates of 96.53% for RNA-Seq data, 99.43% for the MGI data, 99.74% for the PacBio data, and 99.99% for the ONT data. These results collectively indicate high quality of the blackhead seabream genome assembly. Moreover, a BUSCO analysis was performed to assess the completeness of the gene structure annotation, revealing that 96.2% of our annotated genes were correctly classified as complete, with 0.8% being fragmented.

**Table 5:** Statistics of BUSCO evaluation results of previously published^6^ and T2T genome assembly.

Type	Genome (2018)^6^		Genome (T2T)	
	Number	Percentage (%)	Number	Percentage (%)
Complete BUSCOs	3344	91.9	3616	99.3
Complete and single-copy BUSCOs	3307	90.9	3609	99.1
Complete and duplicated BUSCOs	37	1	7	0.2
Fragmented BUSCOs	22	0.6	20	0.6
Missing BUSCOs	274	7.5	4	0.1
Total BUSCO groups searched	3640	100	3640	100

### Collinearity analysis

**Fig. 3:**
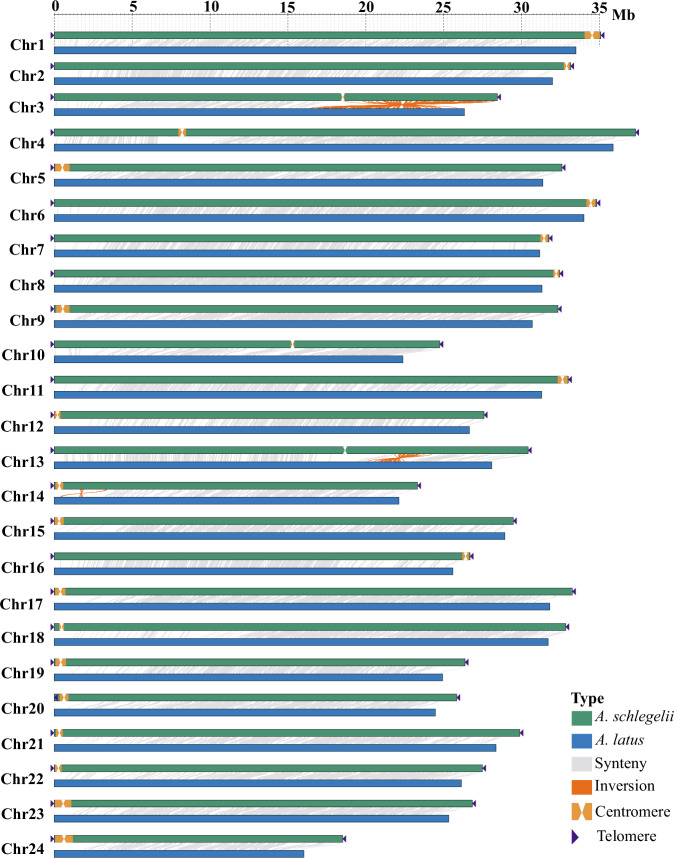
Good synteny of chromosomes between blackhead seabream (*Acanthopagrus schlegelii*) and its relative yellowfin seabream (*Acanthopagrus latus*)^46^.

Whole-genome synteny analysis was performed using the GenomeSyn (v1.2.7)^49^ by aligning the chromosome-level genome assemblies between blackhead seabream (this study) and its relative yellowfin seabream (*A. latus*)^50^. Our results prove that they had excellent one-to-one correspondences among their chromosomes (Fig. 3). This good similarity also underlines the high quality of our sequencing and assembly of the blackhead seabream genome.

## Data Availability

No custom code was used for this study. The versions and parameters for employed software have been deposited in Figshare (10.6084/m9.figshare.26362411.v5). Whenever particular parameters were missing for a software type, the default settings suggested by the creators took effect.
